# **Can dual active ingredient Interceptor**®** G2 insecticide-treated net (ITN) replace indoor residual spraying (IRS) efficiently? A case study in Sakassou, Côte d’Ivoire**

**DOI:** 10.1186/s13071-026-07459-1

**Published:** 2026-05-28

**Authors:** Joseph Chabi, Gloria Salome Shirima, Brian Masanja, Sylvester Coleman, Constant Guy N’Guessan Gbalegba, Bernard Loukou Kouassi, Brice Renaud N’Guessan Broudje, Constant Victorien Ako Edi, Firmain N’Dri Yokoly, William Olatondji Adimi, Ruth-Marie Adjoua Kouame, Valentin Anian, Rosina Kyerematen, Alexander Egyir-Yawson, Samson Kiware, Samuel Kweku Dadzie

**Affiliations:** 1 Abt Global, U.S. President’s Malaria Initiative Evolve Project, Washington, DC USA; 2 Environmental Health and Ecological Science Department, Ifakara Health Institute, P.O. Box 78373, Ifakara, Tanzania; 3 Computational and Communication Science and Engineering, The Nelson Mandela African Institution of Science and Technology, (NM-AIST), P.O. Box 447, Tengeru, Arusha, Tanzania; 4 Department of Vector Biology, Liverpool School of Tropical Medicine, Liverpool, UK; 5 Vector Control Unit, National Malaria Control Programme, Abidjan, Côte d’Ivoire; 6 Abt Global, U.S. President’s Malaria Initiative Evolve Project, Abidjan, Côte d’Ivoire; 7 Swiss Center of Scientific Research in Côte d’Ivoire, Abidjan, Côte d’Ivoire; 8 University Nangui Abrogoua, Abidjan, Côte d’Ivoire; 9 Health District of Sakassou, Regional Health Directorate of Gbêke, Sakassou, Côte d’Ivoire; 10 African Regional Postgraduate Programme in Insect Science (ARPPIS), University of Ghana, Legon, Accra, Ghana; 11 Department of Biomedical Science, University of Cape Coast, Cape Coast, Ghana; 12 Vector Research Group, Department of Parasitology, Noguchi Memorial Institute for Medical Research, University of Ghana, Legon, Accra, Ghana

**Keywords:** IRS, PY-ITN, IG2 ITN, Human biting rate, Entomological inoculation rate, Indoor resting density, Malaria transmission, Malaria cases and incidence

## Abstract

**Background:**

Following three successful rounds of indoor residual spraying (IRS) implementation in the district of Sakassou (Côte d’Ivoire), IRS was withdrawn and replaced by insecticide-treated nets (ITNs). This study evaluated the entomological and epidemiological impacts of Interceptor (IG2) ITNs distributed in Sakassou to determine whether the protection offered by IG2 ITNs was adequate to suppress malaria transmission post-IRS withdrawal.

**Methods:**

This study is a quasi-experimental evaluation using historical data and additional data collections on entomological indicators and malaria incidence to assess malaria transmission trends in Sakassou. The vector control optimization model was adapted to evaluate the effectiveness of the IRS and IG2 ITN deployment on malaria transmission dynamics. Additionally, we used an interrupted time series model to analyze routinely reported malaria cases in the District Health Information Management System (DHIS2) to determine the epidemiological impact of both interventions. Counterfactual trends were generated for the post-IRS withdrawal period during which IG2 ITNs were distributed.

**Results:**

The results showed a 55.4% (95% CI 48.3–62.4%) reduction in the human biting rate (HBR) when IRS was deployed and 48.8% (95% CI 42.8–54.6%) when IG2 ITNs were distributed, compared with standard pyrethroid-only nets. No statistical difference was recorded between the HBR of IG2 ITNs and IRS (*P* = 0.164) during the implementation of IG2 ITNs and the counterfactual of IRS. Similarly, IRS resulted in a 64.7% (95% CI 56.6–72.8%) decline in EIR while IG2 ITNs resulted in a comparable reduction of 61.9% (95% CI 54.2–69.6% ;*P* = 0.616) over the same period. Furthermore, a 26% reduction in malaria cases was recorded immediately after spraying (IRR = 1.02; 95% CI 1.00–1.04; *P* = 0.005) with cumulative impact over time and spray rounds and was similar to IG2 performance (IRR = 1.03; CI 1.00–1.07; *P* = 0.040).

**Conclusions:**

The study findings suggest that IG2 ITNs provided entomological efficacy comparable to clothianidin-based IRS but could not adequately suppress malaria cases after IRS withdrawal, possibly due to plastic-vector feeding behavior and late timing of IG2 deployment. Overall, the study shows how the dynamics of malaria transmission and operational decisions could impact the effectiveness of both IRS and ITNs as vector control tools. These findings provide key information for malaria programs and policymakers to consider when deploying vector control interventions as countries work toward malaria elimination.

**Graphical Abstract:**

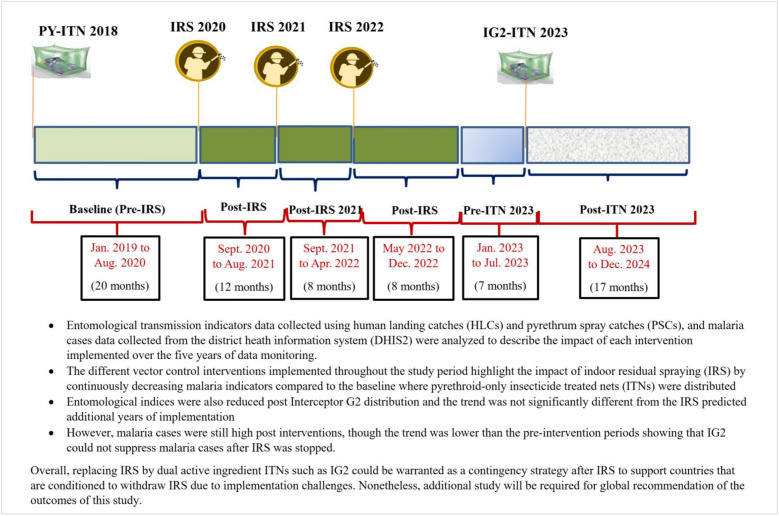

**Supplementary Information:**

The online version contains supplementary material available at 10.1186/s13071-026-07459-1.

## Background

Malaria vector control has historically contributed the most to the declines in malaria burden in sub-Saharan Africa, especially in the last two decades [1]. In most endemic countries, mass distribution of insecticide-treated nets (ITNs) and indoor residual spraying of insecticides (IRS) [2, 3] have been the main vector control interventions used by the malaria programs. Since these interventions rely heavily on the efficacy of the insecticides used, the emergence of vector resistance to available public health insecticides remains a significant threat to their effectiveness and has contributed to the increasing malaria burden [4–7]. It was reported that about 90% of the vectors in endemic countries were resistant to at least one of the pyrethroids used in the impregnation of ITNs [8]. In response to increasing insecticide resistance, malaria control programs have shifted to new-generation vector control products to maintain program efficacy. These include ITNs impregnated with an insecticide and a synergist, such as pyrethroid-piperonyl butoxide (PY-PBO), or with dual active ingredients, such as pyrethroid-chlorfenapyr (PY-CFP). Similarly, for IRS, recently prequalified IRS products such as clothianidin-based insecticides (SumiShield and Fludora Fusion) and broflanilide (Vectron T500) have been introduced. However, this transition to these new products has huge financial implications because they are considerably more expensive than the previously used pyrethroid-only based products [9]. It is estimated that IRS with the new insecticides cost about five times more per person protected per year compared with PY-PBO or PY-CFP ITNs [10]. As a result, ITNs have been prioritized in most countries across Africa and are distributed more widely than IRS. In places where IRS remain part of the malaria vector control strategy it is only selectively deployed in regions with the highest burden. This has affected the sustainability of IRS in many countries, leading to reductions in the geographic coverage of IRS since 2010, facilitated by the decline in funding [11]. Between 2017 and 2025, the number of countries supported by the U.S. President’s Malaria Initiative (one major IRS implementing partner) decreased from 17 to 6 countries [12]. Despite the challenges with IRS, the strategy remains one of the best malaria vector control tools [13, 14]. In places where IRS has been withdrawn or scaled back, its withdrawal has often been linked to a resurgence of malaria, with studies showing increased entomological indices of transmission and malaria incidence rates returning to preintervention levels [15–18].

New-generation ITNs have been suggested as cost-effective alternatives to IRS to sustain the gains of IRS when it is withdrawn, but there are limited data to support this assumption. PBO-ITNs (incorporating a synergist that enhances the insecticide susceptibility of vector populations) were the first new generation of ITNs to be introduced. A meta-analysis on their efficacy in Africa predicted that compared with pyrethroid-only (PY) ITNs, PBO-ITNs could avert up 501 (95% CI 319–621) cases per 1000 people per year in places where there was high pyrethroid resistance [4]. However, follow-up studies have shown that despite their efficacy, the effectiveness of PBO-ITNs often waned significantly after 2 years, even though they are designed to last 3 years [19, 20]. Interceptor® G2 (IG2) is a first-in-class dual active ingredient ITN that combines two different insecticides (chlorfenapyr & alpha-cypermethrin) were also recently prequalified [21]. Studies have shown remarkable efficacy of IG2 ITNs against malaria transmission across the continent, including Côte d’Ivoire, with a much-sustained efficacy against malaria incidence over the duration of its 3-year life span when compared with PBO-ITNs [22–24]. The overall performance of IG2 ITNs has led to it being recommended for use in many countries challenged by high pyrethroid resistance and as a replacement for IRS.

In Côte d’Ivoire, the National Malaria Control Programme (NMCP) and partners supported the implementation of three consecutive IRS campaigns from 2020 to 2022, recording a remarkable reduction of malaria transmission in an area with intense pyrethroid resistance [25–27]. Nonetheless, the strategy had to be discontinued due to the cost of implementation and the need to prioritize resources to fund the scale up of new generation ITNs across the country to protect the entire population at risk. Following the IRS withdrawal, the population of Sakassou were provided with IG2 ITNs. The decision to distribute IG2 ITN post IRS was mainly based on the entomological efficacy of IG2 ITNs that was reported from several studies [24, 28]. However, there are limited data to suggest that IG2 ITNs can provide the protection needed when IRS is withdrawn from an area where IRS has had significant impact on malaria transmission and cases. This study was therefore conducted to assess the entomological and epidemiological impact of IG2 ITNs in a district where IRS was withdrawn and determine whether IG2 ITNs could suppress malaria transmission following the withdrawal of IRS in a highly endemic setting.

## Methods

### Study type

**Fig 1.**
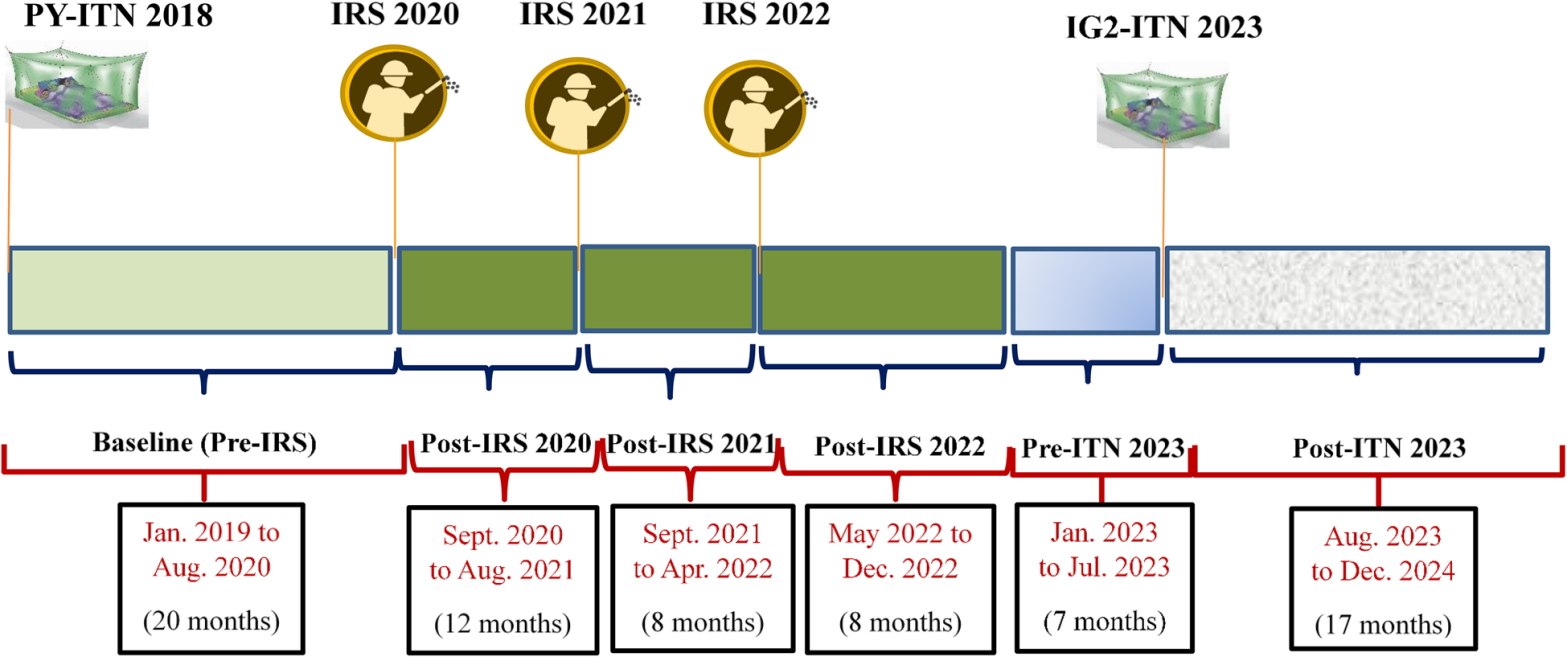
Timeline for the introduction of the different vector control interventions

This study is a quasi-experimental study that combined entomological surveillance and malaria case data with additional prospective entomological collections to assess malaria transmission trends at the study site. Key indicators included vector species and human biting rates (HBR), indoor resting density (IRD), entomological inoculation rate (EIR), and malaria incidence. As interventions were implemented programmatically without randomization, the evaluation followed a before-and-after observational approach. We modeled the impact of sequential interventions across defined implementation periods: (i) PY-ITN considered as baseline from January 2019 to July 2020; (ii) three rounds of IRS and subsequent post-IRS period from August 2020 to July 2023; and (iii) IG2 ITN distribution and post evaluation from August 2023 to December 2024 (Fig. 1).

### Study site

**Fig 2.**
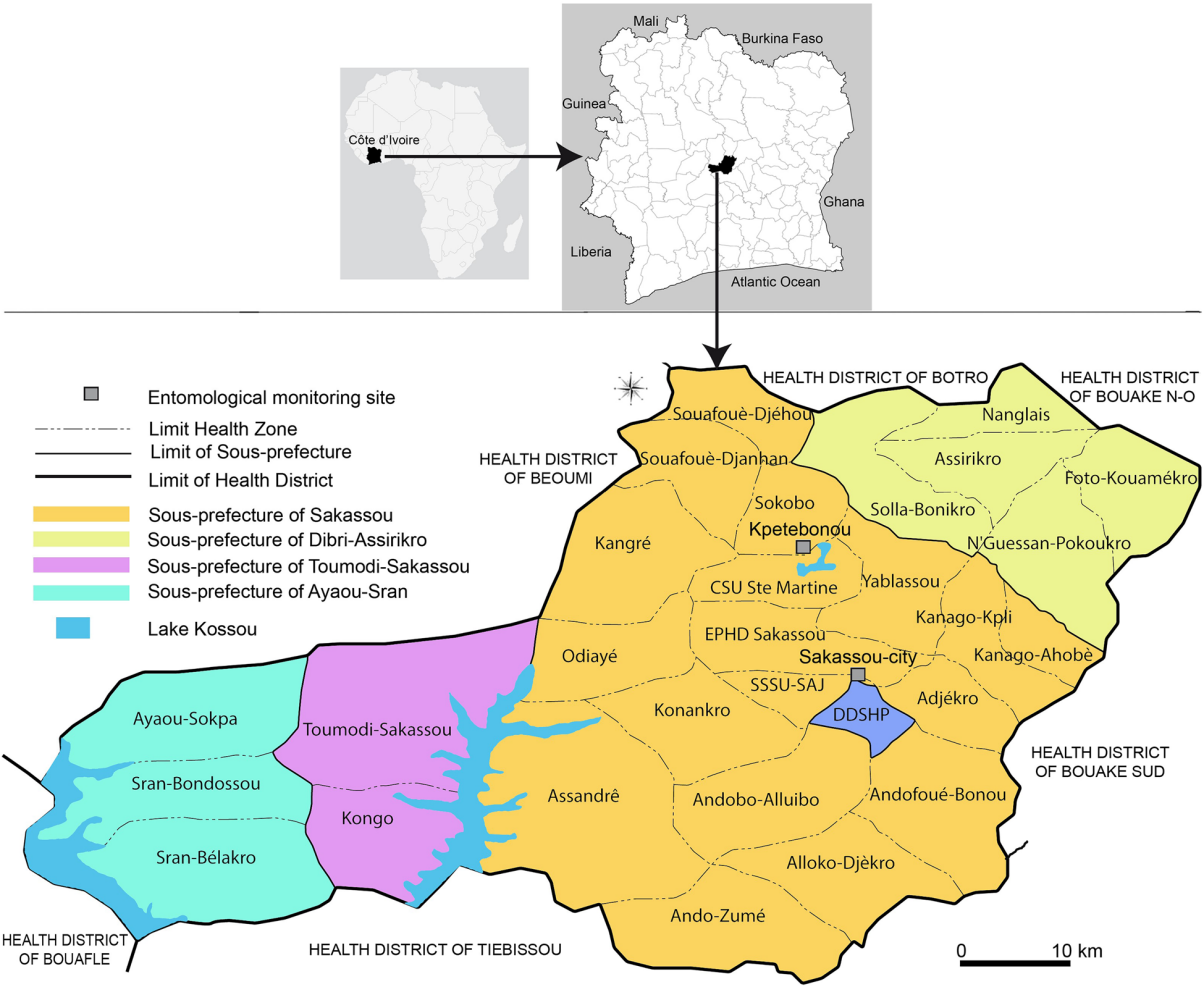
Map of the district of Sakassou showing the 2 entomological monitoring sites and 28 health zones from where epidemiological data were collected

The study was conducted in Sakassou district, situated in the pre-forested savannah zone covering an area of 1820 km^2^. The climate is tropical with two rainy seasons (March–June and September–October) and two dry seasons (July–August and November–February). However, in recent years, rainfall has varied due to climate change. The average annual rainfall is about 899.6 mm, and the mean annual temperature is 26 °C. Sakassou district has 172 villages and hamlets and 28 health facilities that serve a population of about 126,470 inhabitants (Fig. 2). The district recorded the highest malaria incidence in Côte d’Ivoire between 2015 and 2020, according to the NMCP [29].

### Vector control interventions

As per the NMCP’s national strategic plan (NSP) for the period 2016–2021, the country conducted its third ITN mass distribution in 2018 where PY-ITNs were distributed in Sakassou. Afterward, IRS was implemented once a year from 2020 to 2022 with a mean annual coverage of 95% and 128,817 inhabitants protected [26] over the three consecutive years of implementation. In 2023, the NMCP carried out a mini-ITN distribution campaign focused solely on IRS sites, following IRS withdrawal. The targeted campaign successfully reached high operational coverage in Sakassou, with 96% of the planned ITNs distributed[30].

### Entomological data collection

Human landing catches (HLCs) and pyrethrum spray catches (PSCs) were conducted every month in Sakassou before, during, and after IRS and introduction of IG2 ITNs from January 2019 to December 2024. HLCs were carried out in four houses and PSCs in 30 houses during two consecutive days each month to capture any seasonality related to vector density and behavior. HLCs were conducted in the same houses throughout the study period. For PSCs, the houses were selected each month a night prior to pyrethrum spraying within the community and were different from HLC houses. The households available at each collection month and accepting the spraying were considered for PSCs.

HLCs were performed inside and outside of the four houses from 6:00 p.m. to 6:00 a.m. A team of 16 volunteer mosquito collectors from whom written consents were received (2 teams of 8 mosquito collectors, each working half night shifts) conducted HLCs every month.

Mosquitoes collected were morphologically identified to species or species complex using standard identification keys [31]. All malaria vectors were preserved on silica gel in Eppendorf tubes and stored for molecular identification of sibling species and determination of *Plasmodium* infection status. Genomic DNA was extracted from the legs and wings of individual anopheles mosquitoes using the LIVAK method [32] and processed by polymerase chain reactions (PCR). Members of the *Anopheles* (*An*). *gambiae* s.l. complex species (*An. gambiae* s.s., *An. coluzzii*, and *An. arabiensis*) were identified by short interspersed element (SINE)-PCR [33].

To detect the presence of *Plasmodium* parasites, the head and thorax of *An. gambiae* s.l. and *An. funestus* s.l. collected using HLC were analyzed by enzyme-linked immunosorbent assay (ELISA) to detect circumsporozoite proteins as described by Wirtz et al. [34]. This method uses a monoclonal antibody that recognizes a repetitive epitope on the circumsporozoite protein of *Plasmodium falciparum*. ELISA Reagent Kits (MRA-890) were obtained from BEI Resources (NIAID, NIH, USA). Examination of assayed samples was done after reading optical densities (OD) at 405 nm on an ELISA plate reader (Biotek ELx800, Swindon, UK). Positive samples were determined by OD readings twofold greater than the negative controls and were tested a second time for validation of all positive samples detected during the first test using the boiling method [35].

### Epidemiological data collection

Monthly confirmed malaria case data of 28 health facilities (Fig. 2), was retrieved from the health management information system (HMIS) for this analysis. Health facility data were extracted from consultation registers at all health facilities of the district. For each patient recorded in the registers, the rapid diagnostic test (RDT) result and microscopy result were recorded for the period of the study. A confirmed malaria case was defined as any positive RDT or microscopy result.

## Statistical analysis and data interpretation

### Modeling entomological impact of the different interventions

The vector control optimization model (VCOM) [36], a computational tool used to predict the impact of combined vector control interventions on mosquito populations, was used in this study to evaluate the impact of the different ITNs and IRS deployment on malaria transmission dynamics [36]. The model incorporates key biological and behavioral processes, including mosquito density, host-seeking behavior, blood-feeding success, resting behavior, and transmission ability. Intervention effects are represented through their impact on mosquito survival and feeding success throughout the mosquito biological processes mentioned. Parameters governing mosquito life-history traits (e.g., mortality rates, biting frequency, and resting behavior) were adapted from the original VCOM framework (Supplementary file S1). Historical entomological and intervention data from Sakassou were used to inform intervention-specific parameters, including probability of mosquito feeding and surviving in presence of ITNs and IRS, coverage levels, and timing of deployment. These data were used to approximate local transmission conditions and guide the selection of parameter values relevant to the study setting. Rainfall was incorporated into the model as a climatic predictor of mosquito abundance. Rainfall was used to modulate larval habitat availability and carrying capacity, thereby influencing mosquito population dynamics and seasonal variation in vector density. For the forward simulations, the model extended the observed rainfall pattern to represent seasonal trends over time. Furthermore, the Monte Carlo Markov Chain (MCMC) method was used to estimate the different parameters of interest.

Simulations were performed using field data to assess the field scenario and also generate counterfactual predictions on the continued deployment of a given intervention to assess its impact over time. The model simulated the effects of three interventions: (i) deployment of PY-ITNs prior to the introduction of IRS from January 2019 to July 2020; (ii) IRS conducted using clothianidin-based insecticides from August 2020 through July 2023; and (iii) deployment of IG2 ITN from August 2023 to December 2024, following IRS. However, the pre-IG2 ITN period was considered as an extended time for the IRS in all analysis. The intervention timelines in the model simulations were designed to mimic implementation scenarios, with specific months designated for the initiation and cessation of each intervention (Fig. 1). The main entomological outcome indices used to assess the entomological efficacy of these interventions were the indoor resting density (IRD; the mean number of female mosquitoes per room), human biting rate (HBR; the mean number bites per person per night), and the entomological inoculation rate (EIR; the mean infected bites per person per night). These indices were used to quantify the relative impact of each intervention on malaria entomological dynamics.

### Interrupted time series analysis of epidemiological data and predictions

Routine surveillance data obtained from health facilities in the Sakassou district, Côte d'Ivoire were aggregated to the district level and structured as a monthly time series from September 2018 onward. The unit of analysis was the district-month. Each observation comprised the total number of confirmed malaria-positive (RDT) cases (positive), the estimated resident population at risk (pop), calendar month, year, and sequential time index (time), in addition to intervention status variables described in the Supplementary file S2.

### Intervention timeline

Four epidemiological phases were defined on the basis of the programmatic history of vector control in the district. Baseline was the period prior to any indoor residual spraying (IRS), during which only standard PY-ITNs were distributed. IRS period was the commencement of IRS, representing the three rounds. IRS withdrawal phase was the gap period between cessation of IRS and introduction of the second-generation insecticide (IG2 ITN), also depicted as pre-IG2 ITN, during which no IRS was active. Lastly, the IG2 ITN period was the introduction of the IG2 ITN, replacing the IRS compounds.

### Statistical modeling framework

A segmented Poisson regression model was initially specified to estimate the impact of each intervention phase on monthly malaria case counts using the natural logarithm of the district population as an offset to model incidence rates. The interrupted time series (ITS) framework included three types of parameters for each intervention transition: (i) a level-change indicator capturing the immediate step change in incidence at the point of transition; (ii) a slope-change variable (time since intervention start) capturing the change in monthly trend following each transition; and (iii) calendar month fixed effects (with January as reference) to control for seasonal variation in malaria transmission. Definitions of variables and their interpretations can be found in Supplementary file S3.

### Model selection and over-dispersion

The initial Poisson model exhibited severe over-dispersion (dispersion ratio ≈ 118; formal over-dispersion test: *z* = 6.0, *P* < 0.001; estimated dispersion = 86.7). A quasi-Poisson model was fitted as a first correction, which retains identical point estimates while inflating standard errors by the square root of the dispersion parameter. A negative binomial (NB) regression model was then fitted as the preferred alternative, as it explicitly models extra-Poisson variation through an estimated dispersion parameter (theta). The NB model yielded a residual deviance of 76.4 on 57 degrees of freedom (dispersion ratio ≈ 1.34; goodness-of-fit *P* = 0.044), indicating substantially improved fit over the standard Poisson specification.

### Residual autocorrelation and heteroskedasticity and autocorrelation consistent (HAC) correction

Given the time-series structure of the data, residual autocorrelation was assessed using the autocorrelation function (ACF) and partial autocorrelation function (PACF) of deviance residuals from the NB model. The PACF revealed significant partial autocorrelation at lags 1 and 2 (indicating a short-term AR(2) process), while both the ACF and PACF showed a significant negative spike at lag 12, indicating residual seasonal autocorrelation not fully absorbed by the month fixed effects.

To account for residual autocorrelation in a single-district time series, Newey–West heteroskedasticity and autocorrelation consistent (HAC) standard errors were applied to the NB model. The optimal bandwidth was selected using the Andrews (1991) automatic bandwidth selection procedure (implemented via *bw*NeweyWest with prewhite = *FALSE*), which returned a bandwidth of 10.7 (approximately 11 lags), consistent with the seasonal autocorrelation structure identified in the ACF. Two sensitivity analyses were also conducted using fixed bandwidths of 2 (motivated by the PACF AR (2) signal) and 12 (motivated by the seasonal lag-12 spike). We fitted a negative binomial GLM with HAC standard errors (Newey–West, auto-bandwidth = 10.7). Results are reported as incidence rate ratios (IRRs) with 95% confidence intervals and two-sided *P*-values. Statistical analyses were performed in R (version 4.5.2), using the MASS, sandwich, *lm*test, and *gt*summary packages.

## Results

### Trends in entomological parameters

#### Indoor resting density (IRD)

**Fig 3.**
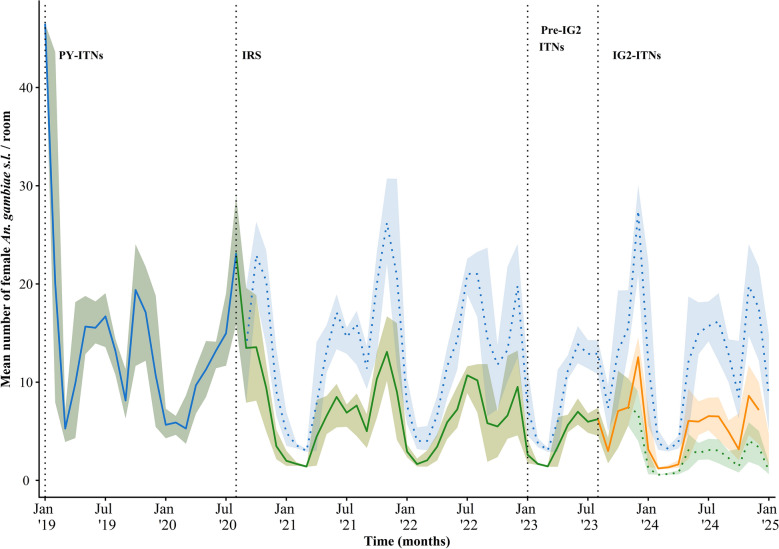
Observed and predicted impact of IRS and IG2 ITNs on the mean IRD of *An*. *gambiae* s.l. from January 2019 to December 2024. The solid line represents fitted values and the dashed line predictions (blue = PY-ITNs deployment; forest green = IRS deployment; dark orange = IG2-ITN deployment). Ribbons represent 95% CI (mean ± 1.96 × SD). The vertical dotted lines delineate the different intervention periods

The analysis showed that IRS resulted in an immediate 52.0% (95% CI 46.5–61.8%) reduction in the IRD of the predominant vector when compared with the PY-ITN period, which was sustained through the period of IRS implementation (Table 1). In comparison with the PY-ITN (pre-IRS) period, IG2 ITNs also resulted in about 45.0% (95% CI 39.9–54.2%) reduction in IRDs. The impact of the counterfactual IRS and IG2 ITN deployment on the IRD of *An. gambiae* s.l. were similar (*P* = 0.169) considering the same period (IRR = 21.28; 95% CI 16.02–28.53) (Fig. 3, Supplementary file S1).

**Table 1:** Summary of final model results

Parameter	IRR	95% CI	*P*-value
PY-ITN intervention (time)	0.985	0.972–0.998	0.026
IRS introduction (level)	1.014	0.826–1.244	0.898
IRS slope (time since IRS)	1.021	1.006–1.036	0.005
IRS withdrawal (level)	1.775	1.497–2.106	< 0.001
Post-IRS withdrawal slope	0.966	0.936–0.996	0.028
IG2 ITN introduction (level)	1.216	1.048–1.412	0.009
IG2 ITN slope (time since IG2 ITN)	1.034	1.002–1.068	0.04
February	0.822	0.749–0.902	< 0.001
March	0.798	0.720–0.883	< 0.001
May	1.197	1.059–1.352	0.004
June	1.222	1.065–1.402	0.004
July	1.259	1.011–1.567	0.038
August	1.355	1.059–1.735	0.015
October	1.215	1.056–1.397	0.006

All results from negative binomial regression with Newey–West HAC standard errors. January was the reference month for seasonal effects

IRR, incidence rate ratio; CI, confidence interval

#### Human biting rate (HBR)

**Fig 4.**
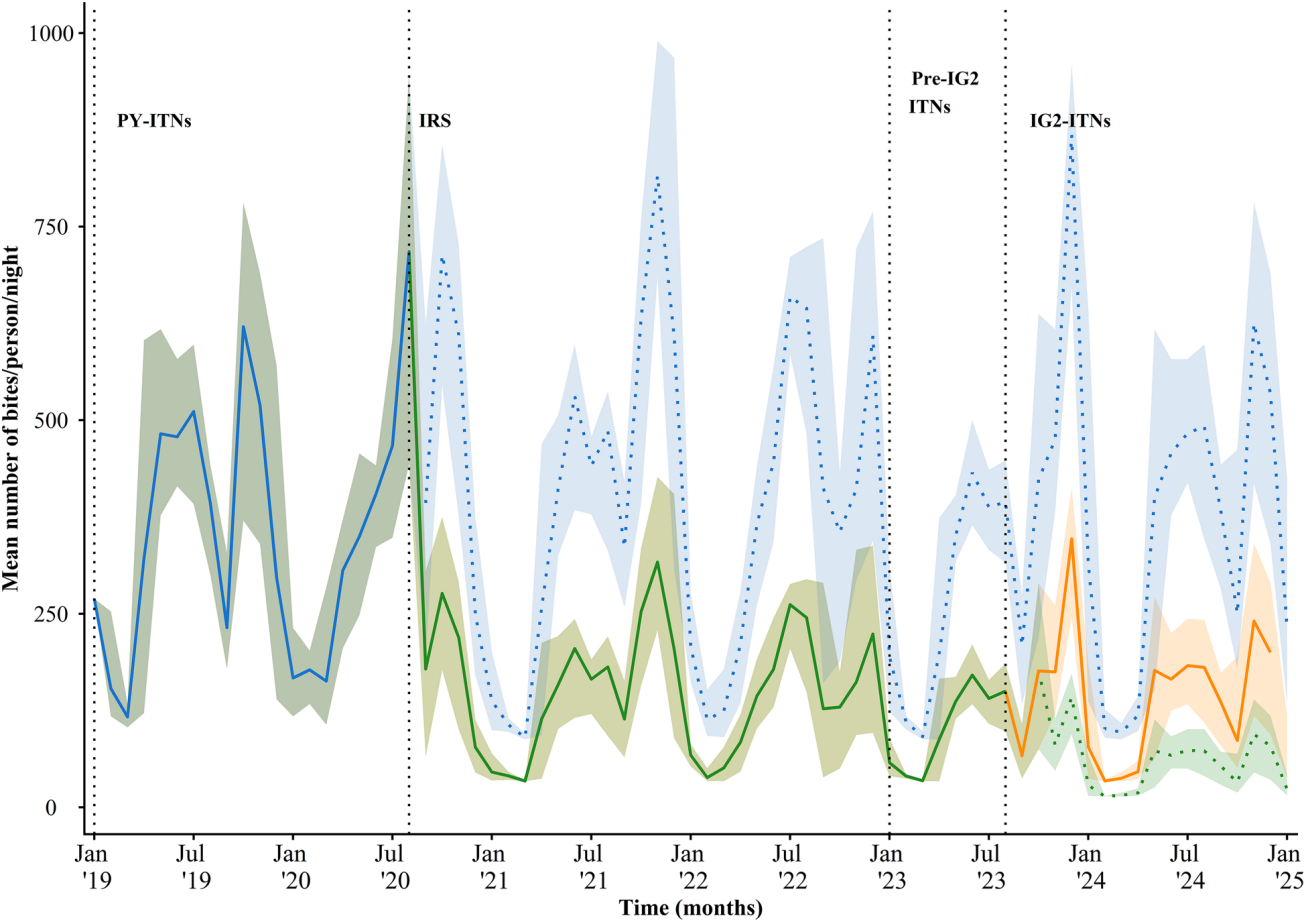
Observed and predicted impact of IRS and IG2 ITNs on mean HBR of *An*. *gambiae* s.l. from January 2019 to December 2024. The solid line represents fitted values and the dashed line predictions (blue = PY-ITNs deployment; forest green = IRS deployment; dark orange = IG2-ITN deployment). Ribbons represent 95% CI (mean ± 1.96 × SD). The vertical dotted lines delineate the different intervention periods

Simulation from the model indicates a greater reduction in human biting rates during the IRS period (55.4%; 95% CI 48.3–62.4%) compared with when IG2 ITNs were deployed (48.8%; 95% CI 42.8–54.7%) with the standard PY-ITNs (Table 1). A counterfactual scenario of continued IRS would have resulted in a further decline in HBR than was observed when IG2 ITNs were deployed (IRR = 19.03; 95% CI 13.06–25.01). However, this projected IRS impact was comparable to the observed effect that the IG2 ITNs had on the HBR of *An. gambiae* s.l. (*P* = 0.164) (Fig. 4, Table 1, Supplementary file S1).

#### Entomological inoculation rate (EIR)

**Fig 5.**
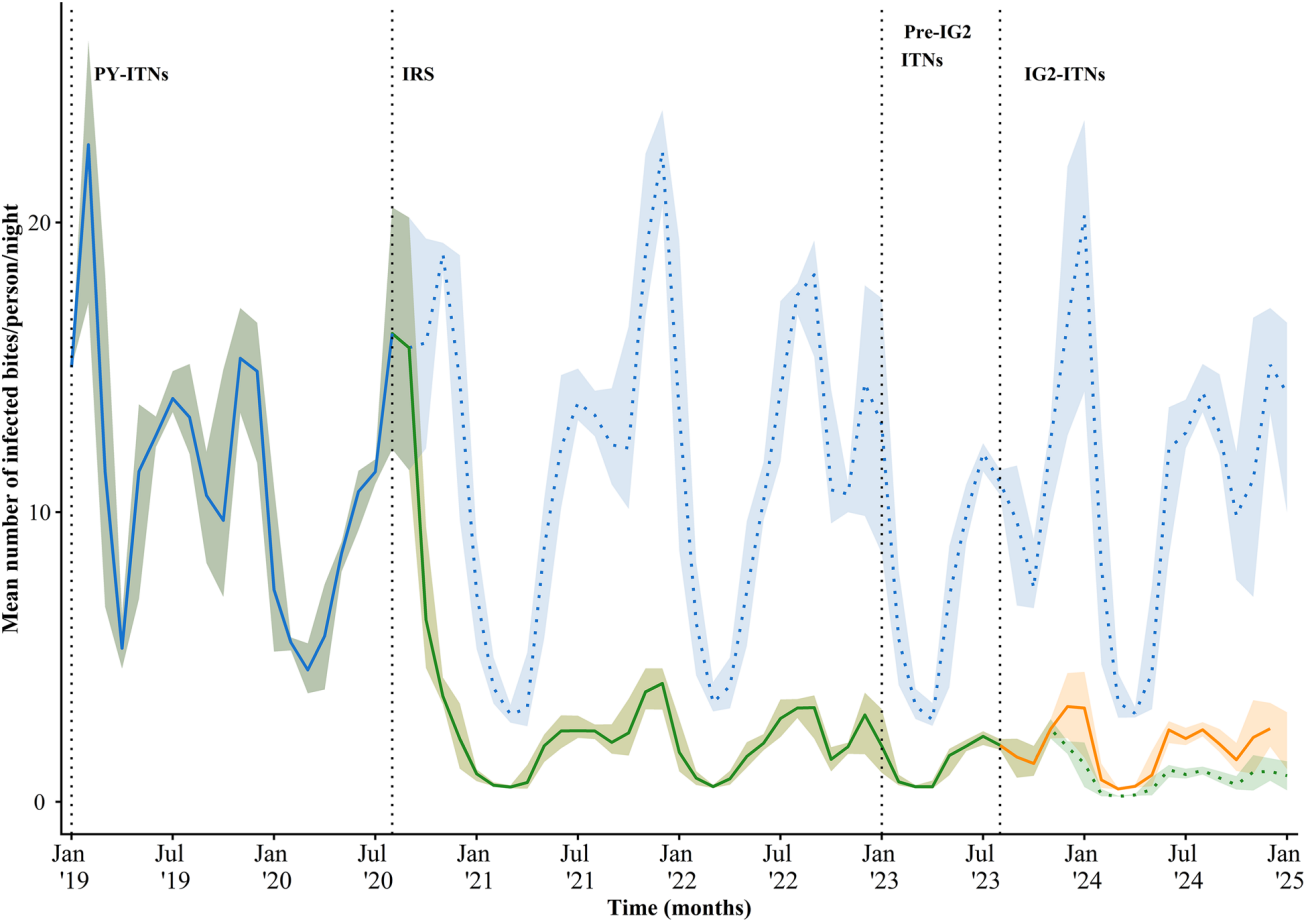
Observed and predicted impact of IRS and IG2 ITNs on mean EIR of *An*. *gambiae* s.l. from January 2019 to December 2024. The solid line represents fitted values and the dashed line predictions (blue = PY-ITNs deployment; forest green = IRS deployment; dark orange = IG2-ITN deployment). Ribbons represent 95% CI (mean ± 1.96 × SD). The vertical dotted lines delineate the different intervention periods

Figure 5 shows observed and predicted EIRs when PY-ITN, IRS, or IG2 was deployed. The analysis indicates that both IRS and IG2 ITNs resulted in significant declines in EIR when compared with the baseline period when PY-ITNs were used. IRS resulted in a 64.7% (95% CI 56.6–72.8%) decline in EIR while IG2 ITNs resulted in a comparable reduction of 61.9% (95% CI 54.2–69.6%; *P* = 0.616) over the same period (IRR = 17.42; 95% CI 11.79–23.05) (Fig. 5, Supplementary file S1).

### Trends in epidemiological parameters

**Fig 6.**
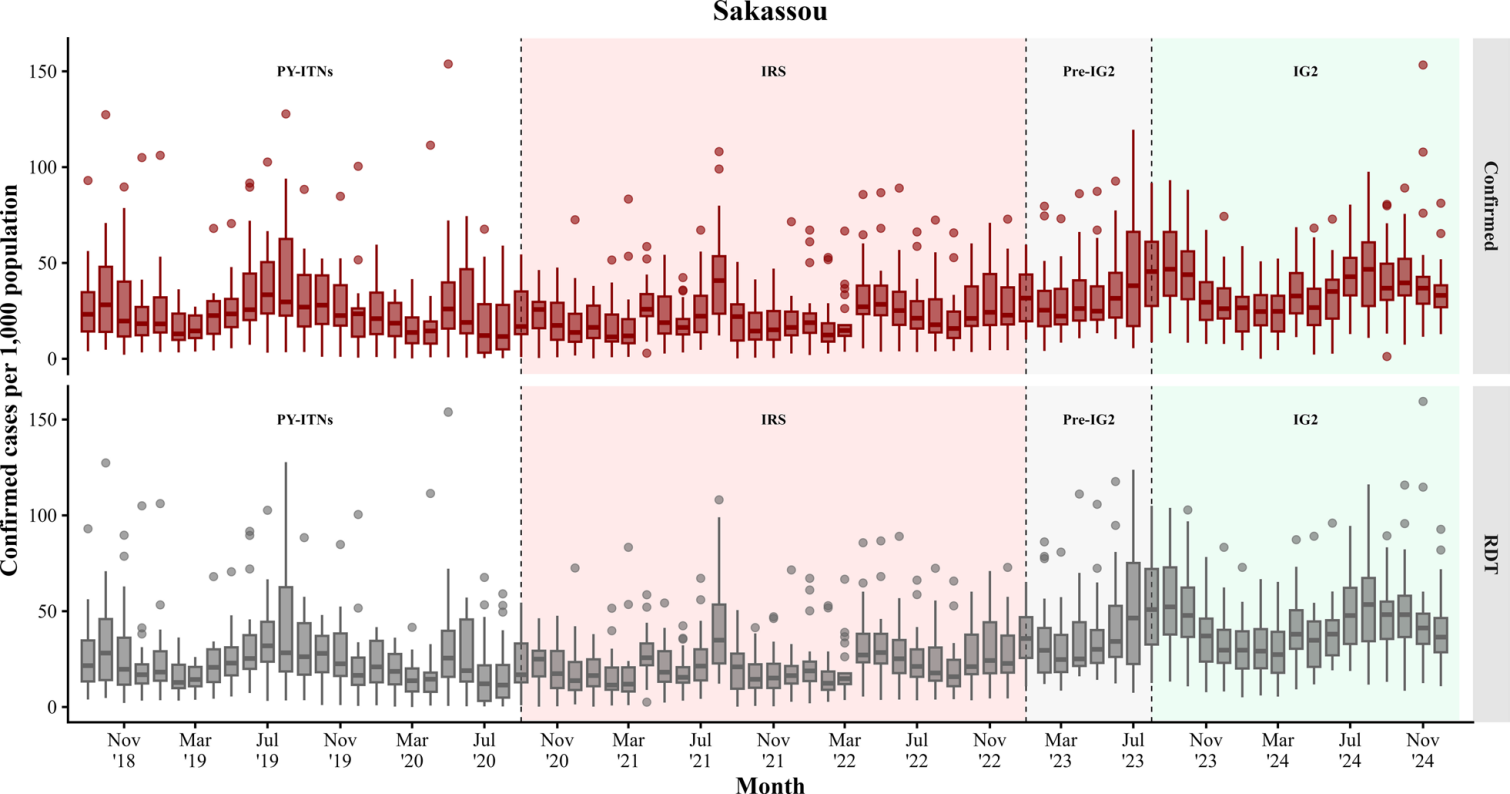
Monthly distribution of facility-level malaria incidence per 1000 population across the four intervention phases in Sakassou district (September 2018–December 2024) for both confirmed and RDT-diagnosed cases

The IRS phase showed broadly comparable median levels with some attenuation of seasonal peaks, particularly in 2021 and 2022. There was a marked elevation in both median incidence and inter-facility spread during the IRS withdrawal phase. However, the extreme values of the withdrawal peak were not sustained following dual active ingredient nets introduction. Incidence remained elevated relative to the IRS phase with persistent inter-facility variability (Fig. 6).

#### Trend prior to IRS intervention

During PY-ITN period and prior to IRS implementation, malaria cases and incidence declined modestly at approximately 1.5% per month (IRR = 0.985; 95% CI 0.972–0.998; *P* = 0.026), indicating a small but statistically significant preexisting trend in the district.

#### Effect of IRS introduction

IRS introduction was not associated with a statistically significant immediate change in malaria incidence at the point of rollout (IRR = 1.014; 95% CI 0.826–1.244; *P* = 0.898). However, a significant upward slope of approximately 2.1% per month was observed during the IRS period (IRR = 1.021; 95% CI 1.006–1.036; *P* = 0.005) as IRS was introduced at a peak transmission period.

#### Effect of IRS withdrawal

**Fig 7.**
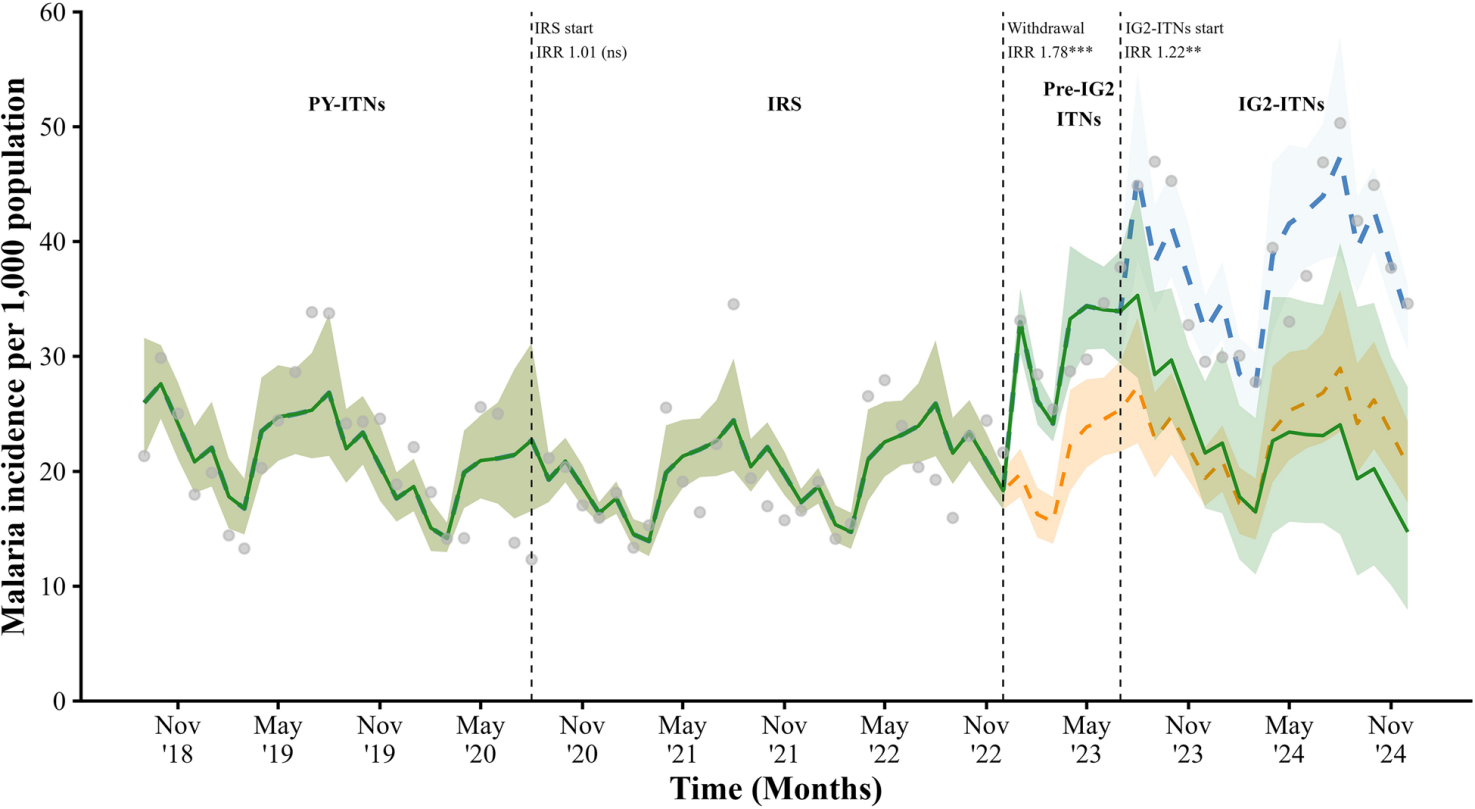
Observed and counterfactual number of malaria incidence in Sakassou prior to the introduction of IRS during the period of IRS implementation and post-IG2 introduction (after IRS withdrawal). The solid line represents fitted values and the dashed line predictions (blue = PY-ITNs deployment; dark orange = IRS deployment; forest green = IG2 deployment). Ribbons represent 95% CI from NB model with Newey–West HAC SD (auto-bandwidth = 1.07). ***P < 0.001, **P < 0.01, ns, significant. The vertical dotted lines delineate the different intervention periods

IRS withdrawal was associated with an immediate and substantially sharp upward step in both the fitted line and observed data in November 2022 after the last round of IRS, with the number of cases and incidence rising from approximately 20 to over 45 per 1000 population within 2 months (IRR = 1.78; *P* < 0.001) (Fig. 7). This finding was consistent across all model specifications tested, including standard Poisson, quasi-Poisson, negative binomial, and NB with Newey–West HAC standard errors at bandwidths of 2, 12, and the auto-selected value of 10.7. Following this immediate surge, incidence declined at approximately 3.4% per month (IRR = 0.966; 95% CI 0.936–0.996; *P* = 0.028). The orange dashed line (IRS continues uninterrupted) projects that, had IRS been maintained, incidence would have remained at approximately 20–25 per 1000 population through 2023–2024.

#### Effect of IG2 ITN introduction

IG2 ITN introduction was associated with an immediate 21.6% increase in malaria cases and incidence (IRR = 1.216; 95% CI 1.048–1.412; *P* = 0.009) with the 95% CI widening progressively, reflecting increased uncertainty over the short post-distribution follow-up period (Fig. 7, Table 1). A modest upward monthly slope of approximately 3.4% per month was also observed after IG2 ITN introduction (IRR = 1.034; 95% CI 1.002–1.068; *P* = 0.040). These IG2 ITN effects reached statistical significance with the auto-bandwidth and lag-12 HAC specifications but were borderline nonsignificant (*P* = 0.075 and *P* = 0.159, respectively), under the more conservative lag-2 specification. The dashed line (withdrawal occurs, but no IG2 ITN is introduced) projects a trajectory intermediate between the IRS-continuation counterfactual and the fitted line during the pre-IG2 phase, converging with the orange counterfactual by mid-2024, suggesting that in the absence of IG2 ITN introduction, the incidence trajectory would have been similar to or lower than the observed fitted values. The overlapping bands of confidence intervals underscore the uncertainty of the projection to warrant any significance of difference.

#### Seasonal pattern

Significant seasonal variation in malaria incidence was observed relative to January, which falls within the long dry season (November–February). Incidence was significantly lower in February (IRR = 0.822; 95% CI 0.749–0.902; *P* < 0.001) and March (IRR = 0.798; 95% CI 0.720–0.883; *P* < 0.001), consistent with the tail of the dry season and the early first rains, respectively, when vector breeding sites have not yet consolidated. Incidence rose progressively from May through August, with significant elevations in May (IRR = 1.197; *P* = 0.004), June (IRR = 1.222; *P* = 0.004), July (IRR = 1.259; *P* = 0.038), and August (IRR = 1.355; *P* = 0.015), reflecting the lagged entomological response to the first rainy season (March–June), with peak transmission persisting into the subsequent dry months as vector populations remain elevated. A second significant elevation in October (IRR = 1.215; *P* = 0.006) corresponds to the second rainy season (September–October), consistent with a bimodal transmission pattern characteristic of the pre-forested savannah zone. The absence of a statistically significant April effect—despite April falling within the first rainy season—likely reflects the lag between rainfall onset and vectorial capacity, with transmission intensity not yet fully established early in the season. These seasonal patterns were robust across all model specifications (Table 1).

## Discussion

This study evaluated the performance of IG2 ITNs in sustaining malaria vector control gains in Sakassou district, Côte d’Ivoire, after IRS was discontinued. Historically, Sakassou has been known to be a high-burden malaria endemic district that contributes the most to the malaria burden in Côte d’Ivoire, with transmission sustained mainly by *An. coluzzii*. Implementation of the IRS with clothianidin-based insecticides resulted in a steady decline in malaria incidence from 15.9% at 1 year after IRS to about 38% by the end of the third round of IRS [25]. This study was designed to determine whether IG2 ITNs could sustain the reductions in malaria transmission indices and suppress malaria cases witnessed during the three rounds of IRS [25, 26]. The findings suggest that IG2 ITNs provided comparable entomological efficacy to counterfactual of IRS continuation. However, within the available observation window, IG2 ITN introduction was not associated with a statistically significant reduction in malaria incidence relative to the post-withdrawal trajectory. All three entomological indicators evaluated (IRD, HBR, and EIR) were comparable between IRS and IG2 ITN deployment periods and IRS counterfactual. However, the counterfactual projections of the malaria incidence data suggest that incidence remained substantially higher than would have been expected if IRS were maintained. These results do not establish IG2 ITN as ineffective; rather, the short post-distribution follow-up period that spanned fewer than two complete seasonal cycles is insufficient to characterize IG2-ITN protective efficacy across a full distribution and net-aging cycle. Extended surveillance is required before conclusions regarding the comparative epidemiological effectiveness of IG2 ITNs relative to IRS can be drawn.

Importantly, IRS was deployed into a population with existing and progressively aging ITN coverage, meaning the model estimated the incremental effect of IRS on top of background net protection rather than the effect of IRS in isolation. The progressive deterioration of the 2018 PY-ITN during the IRS period may have partially offset IRS protective effects at the district level, contributing to the upward slope observed during active spraying. By the time of IRS withdrawal in 2023, the 2018 nets were approximately 4–5 years old and likely beyond functional end-of-life as insecticidal tools, meaning withdrawal of IRS effectively removed the last active insecticidal layer from a population simultaneously experiencing ITN exhaustion. This, in turn, compounds the intervention loss and may help explain the magnitude of the observed incidence rebound.

The counterfactual projections in Fig. 7 provide the clearest visual representation of the central finding of this analysis: the gap between the observed fitted trajectory and the IRS-continuation counterfactual quantifies the epidemiological effect of IRS withdrawal. The projection suggests that maintaining IRS through 2023–2024 would have been associated with incidence remaining at approximately half the levels actually observed following withdrawal, which is a difference of approximately 20–30 cases per 1000 population during peak transmission months. This magnitude is consistent with the burden attributable to the removal of an intervention that had achieved mean coverage of about 95% across three consecutive annual spray rounds, and it reinforces the conclusion that IRS was actively and substantially suppressing transmission throughout its deployment period.

Even though IRS was effective to substantially decrease malaria transmission and incidences, the challenge of cost in the implementation of IRS across malaria-endemic countries is continuously affecting the number of countries that are able to sustain the strategy [14, 37, 38]. The study showed that continuing the implementation of IRS cumulatively reduced malaria vector density and malaria cases in the study area. The observed prediction of 5-year IRS implementation reduced both entomological and epidemiological indicators of malaria in Sakassou. The data analysis showed that the additional years of implementing IRS suppressed the vector density up to 70%, and this resulted in an immediate and significant reduction of approximately 26% of malaria cases in Sakassou. This is consistent with previous studies that showed that IRS was an effective vector control intervention, and the trend is confirmed by several reports from countries such as Ghana, Benin, and Uganda, where IRS was conducted for more than a decade [14, 38–40].

The convergence of the two counterfactual lines by mid-2024 is noteworthy. It suggests that, within the modeled trajectory, the IG2 ITN introduction did not substantially alter the incidence path relative to a scenario in which no replacement intervention had been deployed. This should be interpreted cautiously, because the counterfactual projections are extrapolations beyond the observed data range and carry wide confidence intervals, particularly for the IG2 ITN phase, where fewer than two full seasonal cycles of post-distribution data were available. These projections therefore do not constitute an evidence of IG2-ITN ineffectiveness; rather, they reflect the fundamental limitation that the current observation window is insufficient to detect or exclude a protective effect of IG2 ITNs operating over a complete distribution and aging cycle.

However, the marginal increase (16%) in the number of malaria cases 15 months post-IG2 ITN distribution, though nonsignificant, contrasts with findings from other studies in West and East Africa. In Ghana and in Tanzania, deployment of IG2 ITNs was associated with about a 30% and 44% reduction in malaria incidence, respectively [19, 41], though the comparison factor was PY-ITN rather than IRS. Considering that IG2 ITNs reduced vector biting and transmission intensity, the apparent lack of epidemiological impact observed here may therefore reflect factors unrelated to the intrinsic bioefficacy of IG2 ITNs. The hypothesis is that this could be due to protection gaps resulting from possible human and vector behavior patterns that allow significant human–vector interaction beyond the reach of IG2 ITNs [42–44] or operational decisions that resulted in suboptimal protection. Furthermore, residual outdoor transmission could be considered as one of the hypothesis for maintaining malaria transmission in area of extensive vector control intervention [45, 46]. Additionally, it is possible that the differential expression of specific P450 enzymes by the malaria vectors known to convert chlorfenapyr to its toxic metabolite [47] could have contributed to the limited impact of IG2 ITNs observed in Sakassou. Variability in the expression of these metabolic enzymes across vector populations can influence the activation and effectiveness of chlorfenapyr-based interventions. Notably, earlier work by Chabi et al. [48] reported nonsignificant CYP6P3 expression in *An. coluzzii* populations from this area, suggesting reduced metabolic activation of chlorfenapyr and potentially diminished efficacy of IG2 ITNs.

*An. coluzzii* was reported to feed equally indoors and outdoors in Sakassou [26]. This plastic feeding or a gradual shift to outdoor biting after three rounds of IRS (with deltamethrin and clothianidin), coupled with human night-time outdoor activities, may have led to significant exposure even when people were presumed protected by ITNs [49]. Increased insecticide pressure from the scale-up of ITNs has been shown to increase exophagy and exophily and this can sustain malaria transmission outdoors [50]. Findings from the previous semi-field trials in M'bé, Côte d'Ivoire showed that IG2 ITNs induced deterrence and exit rates up to about 50%. In Tanzania and Uganda, 12% and 49% of the malaria transmission, respectively, occurred before sleeping time [51]. Additionally, in places where individuals spend significant times outdoors between evening and early morning, either sleeping outdoors (without ITNs) or involved in nighttime outdoor activities without any personal protection, there is a high risk of acquiring malaria [43, 52, 53]. In such instances, additional messaging on correct and sustained use, as well as the use of additional personal protection and larval source management, may be warranted to complement indoor targeted interventions. Additionally, the timing of the deployment of vector control interventions also plays a key role in their epidemiological impact. The optimal time of year for deployment of interventions is before the transmission season [54].

Our analysis indicates that malaria transmission is perennial in Sakassou and highly variable by month, with peaks that typically coincide with the long rainy season (March–July) and the end of the short rainy season (September–November) [54]. Therefore, the optimal timing for deployment of ITNs would have been in March/April 2023. However, the IG2 TNs were distributed in August 2023, during the peak of the malaria transmission season, which began in July 2023. In addition, there were about 15 months between the last IRS campaign in May 2022 and the distribution of IG2 ITNs in August 2023, which could have affected the malaria case trends. By March 2023, 10 months after the third year’s IRS, which was beyond the residual life of the sprayed insecticide (SumiShield) [55–57], we had begun to see a slight rise in malaria cases. Starting interventions late into the transmission season not only increases the risk of widespread transmission, but also amplifies the infectious reservoir, resulting in suboptimal protection. Findings from a study in Wajir County in northeast Kenya highlight this risk and confirm that the timing of vector control interventions is critical for determining malaria outcomes in settings where malaria transmission is seasonal [58]. The authors reported that the delayed deployment of vector control interventions (6 months after heavy rainfall and flooding) in Wajir county in 1998 was associated with a large and explosive malaria epidemic (with weekly incidence rates peaking at 54/1000 population/week). In contrast, no epidemic was recorded in 2007 when vector control interventions were deployed within 3 months of the start of the rainy season. Weekly malaria incidence rates never exceeded 0.5 per 1000 population. The deployment of IG2 ITNs at the peak of the malaria transmission season in our study district may account for the marginal increase in malaria cases.

Despite that marginal increase in malaria cases following IRS withdrawal, IG2 ITN deployment appears far more effective than the counterfactual scenario of deploying PY-only ITNs, which would have led to a 34% increase in cases, as previously reported in several countries where IRS was stopped [59, 60]. Conversely, additional years of implementing IRS deployed at the right time could have suppressed vector density by up to 70% and resulted in an additional 26% reduction in malaria cases in Sakassou. This is consistent with previous studies from Benin, Ghana, and Uganda (where IRS was conducted for more than a decade) [14, 38–40]. These studies showed that repeated IRS with the right insecticide deployed at the right time could result in significant and sustained entomological and epidemiological impacts.

However, operational adjustments such as improving timing and user education may result in a greater epidemiological impact than was observed. Still, it will be important to continue monitoring the impact of IG2 ITNs over time, and additional studies in different malaria transmission settings will be required to generate further evidence on whether IG2 ITNs can replace IRS or help sustain the gains.

The IG2 ITN model may require additional parameters that were not included in the model, and suggests that either the intervention variable did not capture a true change in exposure or that other confounding factors, such as population behavior related to ITN use or vectors’ response toward the insecticide in the ITNs, malaria case management, and commodities stockouts, may have diluted the signal. Furthermore, the study did not incorporate a control site. Including a control could have enhanced the statistical power of the data and deepened the understanding of the epidemiological information that was presented. Further investigations are needed to clarify whether this represents a true null effect or challenge with data/measurement.

The malaria incidence analysis relies entirely on routine health facility surveillance data aggregated at the district level, which are subject to well-documented limitations, including variation in health-seeking behavior, changes in testing rates and diagnostic practices over time, facility reporting completeness, and shifts in catchment population boundaries, all of which may introduce bias that cannot be fully disentangled from true intervention effects. The instability of population denominators during the IG2 ITN phase, likely reflecting the addition of new facilities and redistribution of catchment boundaries rather than true demographic change, adds further uncertainty to incidence estimates in that period.

The ITS design assumes that the counterfactual trend in the absence of each intervention would have followed the trajectory established in the preceding phase; concurrent changes in health-seeking behavior, vector resistance, or other malaria control activities coinciding with intervention transitions could therefore confound the estimated effects. The post-IG2 ITN observation window spans fewer than two complete seasonal cycles, which is insufficient to estimate IG2 ITN insecticide-treated net protective efficacy across a full distribution and net aging cycle, given that dual active ingredient net efficacy is known to attenuate with net age and cumulative washing. The borderline goodness-of-fit result (*P* = 0.044) and identification of influential outlying months suggest that episodic events not captured by the model structure, such as rainfall patterns, vector density fluctuations, or ecological changes, may have affected model fit in specific periods. Finally, the analysis estimates the population-level epidemiological impact of interventions at the district level and cannot speak to heterogeneity in coverage, access, or transmission intensity across individual health facility catchments within the district, nor to the entomological mechanisms underlying the observed incidence patterns.

## Conclusions

The study reports that replacing IRS with IG2 ITNs was associated with a marginal but not significant increase in the number of malaria cases in Sakassou and similarly to the malaria transmission indicators such as the IRD, HBR, and EIR. The interrupted time series analysis conducted provides robust evidence that IRS was suppressing malaria transmission in Sakassou district, with the clearest signal arising from the large malaria case rebound following withdrawal. The effectiveness of IG2 ITN could not be definitively established within the available observation window, and its introduction coincided with a period of elevated background transmission. These findings highlight the critical importance of maintaining continuity of IRS campaigns: interruptions in spray coverage, even brief ones, can rapidly negate the epidemiological gains achieved through sustained vector control. Future analyses would benefit from longer post-IG2 ITN follow-up, disaggregation by health facility, and integration of entomological monitoring data to validate the biological plausibility of the estimated effects. Overall, the study shows how the dynamics of malaria transmission and operational decisions could impact the effectiveness of both IRS and ITNs as vector control tools. These findings present key information for malaria programs and policymakers for consideration when deploying vector control interventions as countries work toward malaria elimination.

## Supplementary Information

Additional file 1.

Additional file 2.

Additional file 3.

## Data Availability

Data supporting the main conclusions of this study are included in the manuscript.

## Abbreviations

*An*.: *Anopheles*
CI: Confidence interval
DHIS2: District health information software version 2
EIR: Entomological inoculation rate
ELISA: Enzyme-linked immunosorbent assay
HBR: Human biting rate
HLC: Human landing catch
ITN: Insecticide-treated net
IRD: Indoor resting density
IRS: Indoor residual spraying
NMCP: National Malaria Control Programme
PBO: Piperonyl butoxide
PCR: Polymerase chain reaction
PSC: Pyrethrum spray catch
PY: Pyrethroid
RR: Rate ratio
WHO: World Health Organization
