# Supplementary material for: Can dual active ingredient Interceptor® G2 insecticide-treated net (ITN) replace indoor residual spraying (IRS) efficiently? A case study in Sakassou, Côte d’Ivoire
Source: Parasit Vectors. 2026 May 28;19:304. doi: 10.1186/s13071-026-07459-1 (PMC13410900; doi:10.1186/s13071-026-07459-1)
Supplement: Supplementary file 3 — Additional file 3. [file 13071_2026_7459_MOESM3_ESM.docx]

Supplementary Table S1:

| Parameter | Description | Value | Value Range | Source |
| --- | --- | --- | --- | --- |
| recRate | Human recovery rate | 0.15 | 0.05 - 0.2 | [44] |
| muVcom | Adult mosquito daily mortality in the presence of interventions | 0.315 | 0.1 - 0.5 | estimated |
| muPL | Pupal daily mortality | 0.25 | 0.2 - 0.3 | [44] |
| muLL | Late larval instar daily mortality | 0.035 | 0.03 - 0.05 | [44] |
| muEL | Early larval instar daily mortality | 0.034 | 0.02 - 0.05 | [44] |
| lambdaV | Force of infection in vectors at equilibrium | 0.01337 | 0.008 - 0.03 | [44] |
| durPL | Duration of pupal stage | 0.664 | 0.5 - 3 | [44] |
| durLL | Duration of late instar stage | 6.64 | 5 - 8 | [44] |
| durEL | Duration of early instar stage | 6.64 | 5 - 8 | [44] |
| durEV | Duration of latent period in mosquito (days) | 10 | 8 - 12 | [44] |
| Bh | Transmission efficiency from an infectious mosquito to an uninfected, susceptible human | 0.5 | 0.4 - 0.6 | [44] |
| betaCom | Eggs laid per day by female mosquito in the presence of interventions | 21.19 | 15 - 25 | [44] |
| a_theta | Human biting rate per mosquito in the presence of interventions | 0.3 | 0.05 - 0.5 | [44] |
| 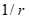 | Average duration of infectiousness in untreated humans | 50 days |  | [1] |
| 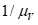 | Average mosquito life expectancy | 8 days |  | [2, 3] |
| 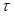 | Average latent period in mosquito host | 10 days |  | [4] |
| 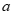 | Biting rate per mosquito on humans | 0.33 per day |  | [5] |
| 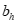 | Vector-to-human transmission probability | 0.2 |  | [6-8] |
| 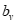 | Human-to-vector transmission probability | 0.05 |  | [9, 10] |
| 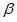 | Number of eggs laid per adult female mosquito per day | 21.19 |  | [11] |
| 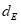 | Developmental period of early instars | 6.64 days |  | [12] |
| 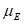 | Death rate of early instars | 0.034 per day |  | [12] |
| 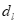 | Developmental period of late instars | 3.72 days |  | [12] |
| 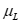 | Death rate of late instars | 0.035 per day |  | [12] |
| 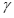 | Density-dependent factor for late instars | 13.25 |  | [11] |
| 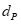 | Developmental period of pupae | 0.64 days |  | [12] |
| 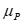 | Death rate of pupae | 0.25 per day |  | [11] |
| 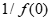 | Reciprocal of gonotrophic cycle length | 0.33 per day |  | [13] |
| 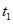 | Mean time spent foraging for a blood-meal | 0.68 days |  | [14] |

| **Parameter:** | **Definition:** | ***An. gambiae*:** | **Reference:** |
| --- | --- | --- | --- |
| 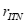 | Probability of repeating a feeding attempt due to PY-ITNs | 0.56  (0.5 – 0.7) | [1] |
| 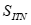 | Probability of feeding and surviving in presence of PY-ITNs | 0.03  (0.03-0.1) | [1, 2] |
| 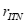 | Probability of repeating a feeding attempt due to IG2-ITNs | 0.56  (0.5 – 0.7) | [1] |
| 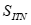 | Probability of feeding and surviving in presence of IG2-ITNs | 0.03  (0.03-0.1) | [1, 2] |
| 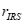 | Probability of repeating a feeding attempt due to IRS | 0.60  (0.5 – 0.7) | [3] |
| 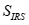 | Probability of feeding and surviving in presence of IRS | 0  (0.02 - 0.1) | [3] |
| 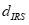 | Mosquito death rate due to IRS | 0.40  (0.35 – 0.5) | [4] |
| 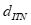 | Mosquito death rate due to ITNs | 0.41  (0.35 – 0.5) | [1, 2] |

The final model analyzing the epidemiological results took the following form:

*log(E[positive_t]) = β₀ + β₁·time + β₂·treatment_IRS + β₃·timeSince_IRS + β₄·withdrawal_IRS + β₅·timeSince_withdrawal + β₆·treatment_IG2 + β₇·timeSince_IG2 + Σ γₘ·monthₘ + log(pop_t)*

**The Poisson model results:**

|  | **IRR** | **95% CI** | **p-value** |
| --- | --- | --- | --- |
| **time** | 0.98 | 0.98, 0.99 | **<0.001** |
| **treatment_IRS** | 1.03 | 1.01, 1.05 | **0.015** |
| **timeSince_IRS** | 1.02 | 1.02, 1.02 | **<0.001** |
| **withdrawal_IRS** | 1.78 | 1.73, 1.84 | **<0.001** |
| **timeSince_withdrawal** | 0.97 | 0.96, 0.97 | **<0.001** |
| **treatment_IG2** | 1.21 | 1.18, 1.24 | **<0.001** |
| **timeSince_IG2** | 1.03 | 1.03, 1.04 | **<0.001** |
| **month** |  |  |  |
| 1 | — | — |  |
| 2 | 0.84 | 0.82, 0.86 | **<0.001** |
| 3 | 0.81 | 0.79, 0.83 | **<0.001** |
| 4 | 1.14 | 1.12, 1.16 | **<0.001** |
| 5 | 1.18 | 1.15, 1.20 | **<0.001** |
| 6 | 1.23 | 1.20, 1.25 | **<0.001** |
| 7 | 1.30 | 1.27, 1.32 | **<0.001** |
| 8 | 1.39 | 1.36, 1.41 | **<0.001** |
| 9 | 1.16 | 1.14, 1.18 | **<0.001** |
| 10 | 1.24 | 1.22, 1.27 | **<0.001** |
| 11 | 1.09 | 1.06, 1.11 | **<0.001** |
| 12 | 0.95 | 0.93, 0.97 | **<0.001** |
| Abbreviations: CI = Confidence Interval, IRR = Incidence Rate Ratio | | | |

**AIC: 7494.8**

Overdispersion test on the Poisson model:

Z = 6.0412, p-value = 7.649e-10.

A p-value lower than 0.05 allows us to reject the null hypothesis that the model is not overdispersed. Clearly there is more variability than the model expects.

**The quasi-Poisson model results:**

| **Characteristic** | **IRR** | **95% CI** | **p-value** |
| --- | --- | --- | --- |
| **time** | 0.98 | 0.97, 1.00 | **0.010** |
| **treatment_IRS** | 1.03 | 0.82, 1.29 | 0.8 |
| **timeSince_IRS** | 1.02 | 1.01, 1.04 | **0.010** |
| **withdrawal_IRS** | 1.78 | 1.26, 2.50 | **0.002** |
| **timeSince_withdrawal** | 0.97 | 0.91, 1.03 | 0.3 |
| **treatment_IG2** | 1.21 | 0.91, 1.61 | 0.2 |
| **timeSince_IG2** | 1.03 | 0.97, 1.10 | 0.3 |
| **month** |  |  |  |
| 1 | — | — |  |
| 2 | 0.84 | 0.67, 1.05 | 0.14 |
| 3 | 0.81 | 0.64, 1.02 | 0.080 |
| 4 | 1.14 | 0.92, 1.41 | 0.2 |
| 5 | 1.18 | 0.95, 1.47 | 0.15 |
| 6 | 1.23 | 0.98, 1.54 | 0.079 |
| 7 | 1.30 | 1.03, 1.63 | **0.029** |
| 8 | 1.39 | 1.12, 1.72 | **0.004** |
| 9 | 1.16 | 0.94, 1.44 | 0.2 |
| 10 | 1.24 | 1.01, 1.53 | **0.048** |
| 11 | 1.09 | 0.88, 1.35 | 0.5 |
| 12 | 0.95 | 0.76, 1.19 | 0.7 |
| Abbreviations: CI = Confidence Interval, IRR = Incidence Rate Ratio | | | |

Another alternative to quasi-Poisson is negative binomial.

**Negative binomial test results:**

| **Characteristic** | **IRR** | **95% CI** | **p-value** |
| --- | --- | --- | --- |
| **time** | 0.98 | 0.97, 1.00 | **0.003** |
| **treatment_IRS** | 1.01 | 0.84, 1.23 | 0.9 |
| **timeSince_IRS** | 1.02 | 1.01, 1.03 | **0.002** |
| **withdrawal_IRS** | 1.78 | 1.28, 2.47 | **<0.001** |
| **timeSince_withdrawal** | 0.97 | 0.90, 1.03 | 0.3 |
| **treatment_IG2** | 1.22 | 0.90, 1.64 | 0.2 |
| **timeSince_IG2** | 1.03 | 0.97, 1.11 | 0.3 |
| **month** |  |  |  |
| 1 | — | — |  |
| 2 | 0.82 | 0.68, 1.00 | **0.045** |
| 3 | 0.80 | 0.66, 0.97 | **0.021** |
| 4 | 1.14 | 0.94, 1.38 | 0.2 |
| 5 | 1.20 | 0.98, 1.46 | 0.075 |
| 6 | 1.22 | 1.00, 1.49 | 0.051 |
| 7 | 1.26 | 1.02, 1.55 | **0.029** |
| 8 | 1.36 | 1.11, 1.65 | **0.003** |
| 9 | 1.14 | 0.94, 1.37 | 0.2 |
| 10 | 1.22 | 1.00, 1.47 | **0.044** |
| 11 | 1.08 | 0.89, 1.30 | 0.4 |
| 12 | 0.94 | 0.78, 1.14 | 0.6 |
| Abbreviations: CI = Confidence Interval, IRR = Incidence Rate Ratio | | | |

**AIC: 1214.3.** The model did better than Poisson by AIC.

Check for residual autocorrelation in the time series models

**ACF plot**


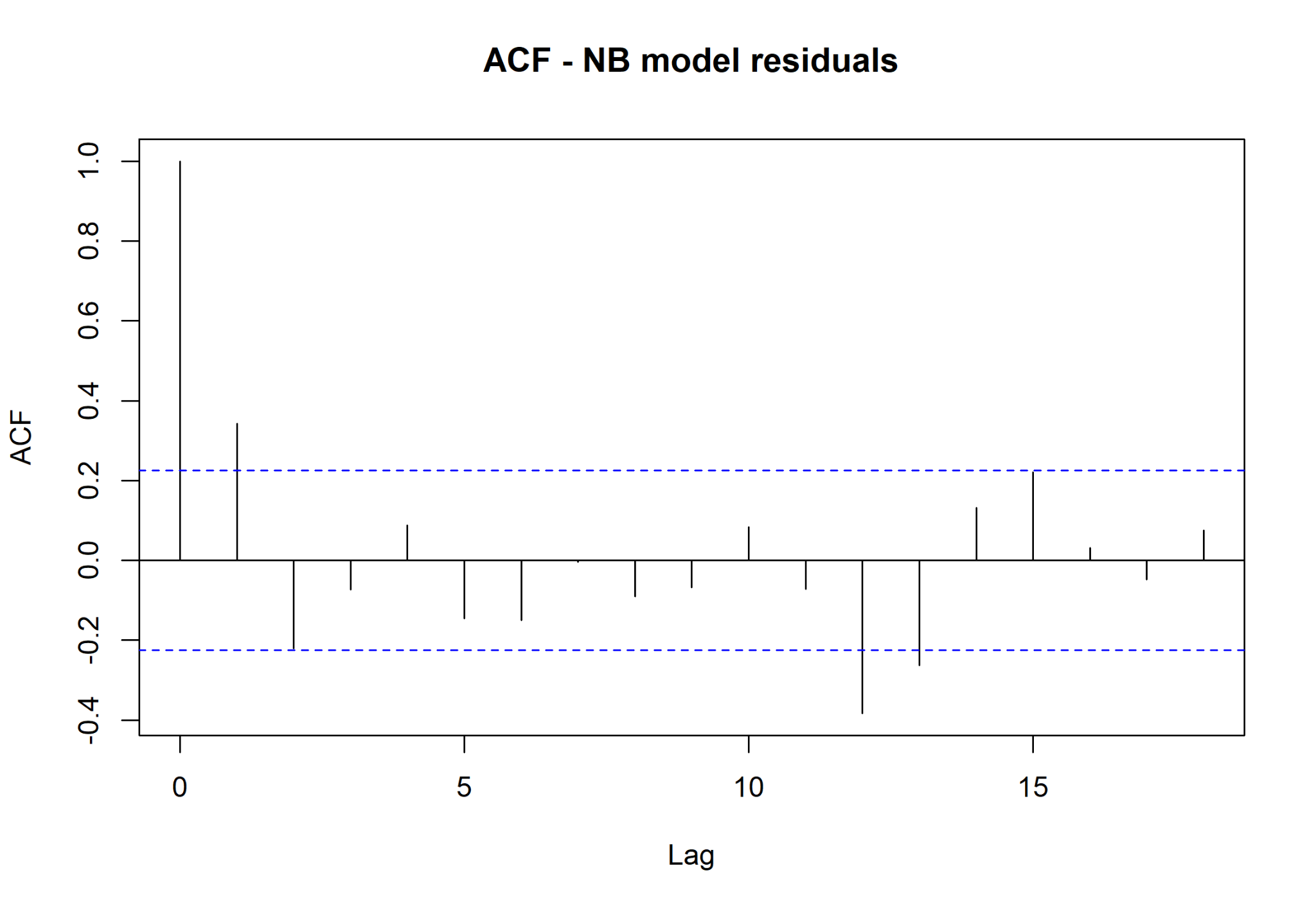


**PACF plot**


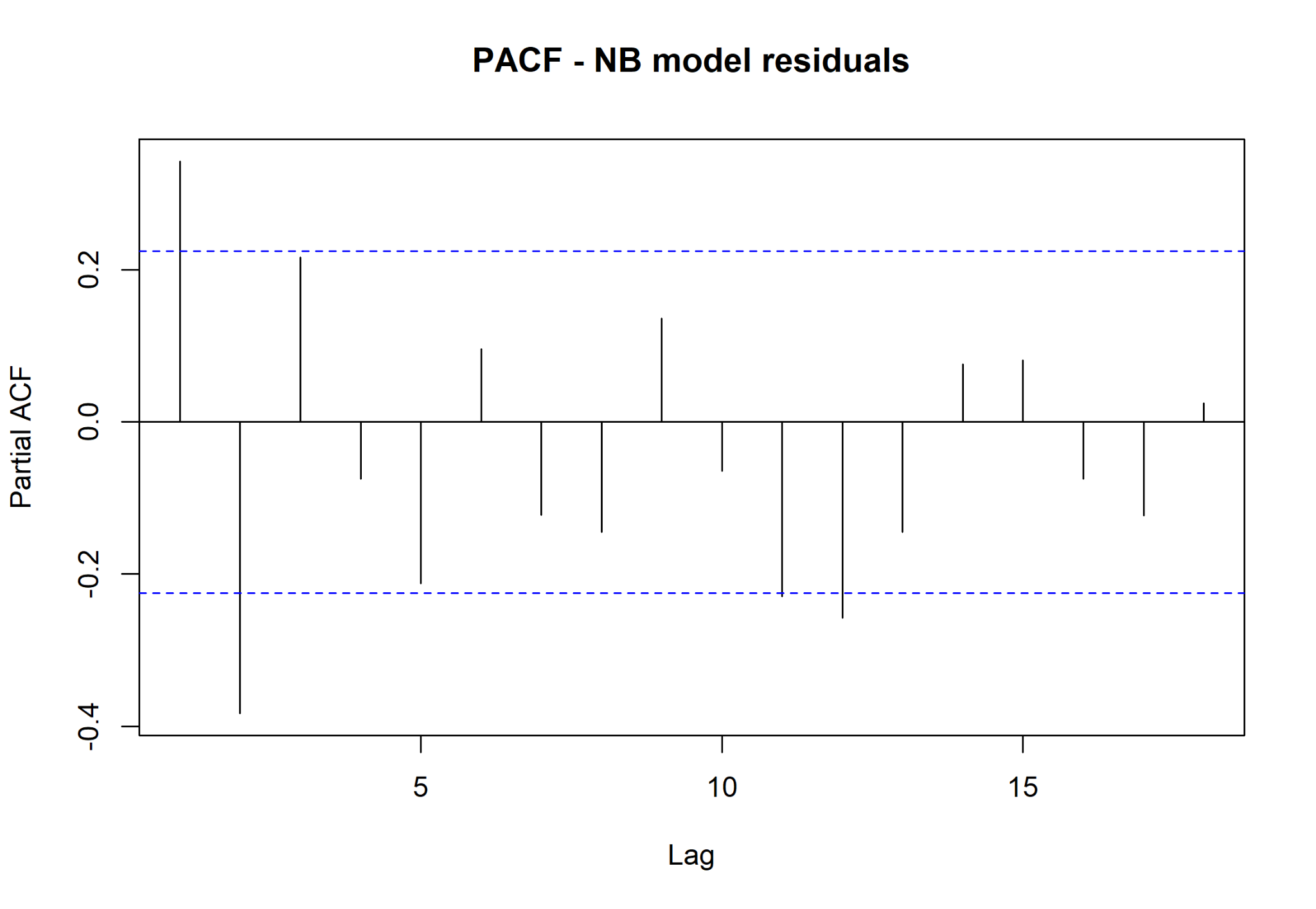


The preferred model in this situation is combining negative binomial with Newey-West HAC, which corrects for heteroscedasticity and serial correlation in time series residuals, which, if ignored, can lead to invalid inference.

A key consideration in this scenario is bandwidth selection, which requires selecting a specified number of lags. Upon inspection of the lags occurring in our time series, it appears we have to select between two: lag 2 and 12; we decided to let the selection be automatic to avoid bias and find an optimum between conservative (lag 2) and seasonal influence (lag 12).
